# What do we manipulate when reminding people of (not) having control? In search of construct validity

**DOI:** 10.3758/s13428-023-02326-8

**Published:** 2024-01-17

**Authors:** Marcin Bukowski, Anna Potoczek, Krystian Barzykowski, Johannes Lautenbacher, Michael Inzlicht

**Affiliations:** 1 Institute of Psychology, Faculty of Philosophy, Jagiellonian University, Kraków, Poland; 2 Interdisciplinary Centre for Mathematical and Computational Modelling, University of Warsaw, Warsaw, Poland; 3 Department of Social Psychology, Leipzig University, Leipzig, Germany; 4 Department of Psychology, University of Toronto, Toronto, Canada; 5 Rotman School of Management, University of Toronto, Toronto, Ontario Canada

**Keywords:** Construct validity, Personal control, Recall-based manipulations, Replicability

## Abstract

The construct of personal control is crucial for understanding a variety of human behaviors. Perceived lack of control affects performance and psychological well-being in diverse contexts – educational, organizational, clinical, and social. Thus, it is important to know to what extent we can rely on the established experimental manipulations of (lack of) control. In this article, we examine the construct validity of recall-based manipulations of control (or lack thereof). Using existing datasets (Study 1a and 1b: *N* = 627 and *N* = 454, respectively) we performed content-based analyses of control experiences induced by two different procedures (free recall and positive events recall). The results indicate low comparability between high and low control conditions in terms of the emotionality of a recalled event, the domain and sphere of control, amongst other differences. In an experimental study that included three types of recall-based control manipulations (Study 2: *N* = 506), we found that the conditions differed not only in emotionality but also in a generalized sense of control. This suggests that different aspects of personal control can be activated, and other constructs evoked, depending on the experimental procedure. We discuss potential sources of variability between control manipulation procedures and propose improvements in practices when using experimental manipulations of sense of control and other psychological constructs.

The construct of personal control has been at the center of psychological science for decades, showing its crucial impact on physical and mental well-being but also on people’s social functioning (Bukowski, Fritsche, et al., 2017; Hong et al., 2021; Skinner, 1996). The notion of personal control refers to the extent to which a person or agent believes that he or she can produce desired outcomes and prevent undesired ones (Skinner, 1996). Experiencing prolonged states of uncontrollability has consistently been shown to carry heavy cognitive, emotional, and motivational costs for humans and animals (Kofta & Sedek, 1998; Mikulincer, 1994; Seligman, 1975).

Interest in the construct of personal control has grown more recently after numerous scholars showed that when people lack a sense of control, they tend to search for ways to compensate by perceiving structure and order in their environment (Friesen et al., 2014; Kay et al., 2008; Landau et al., 2015). Lacking personal control, for example, has been found to lead people to adhere to superstitions, as well as believe in a controlling god, in conspiracies, or even in precognition (Greenaway et al., 2013; Kay et al., 2009; Whitson & Galinsky, 2008). This research carried a promise of revealing something important about human nature, by answering the enduring question of why we hold certain types of beliefs when faced with a threat to personal control.

In the ensuing years, however, some of these findings could not be replicated (e.g., van Elk & Lodder, 2018; Hoogeveen et al., 2018). For example, van Elk and Lodder (2018) examined the effects of control threat manipulations on measures of magical thinking, conspiracy beliefs, paranormal beliefs, and agent detection in seven experiments and could not find any evidence of the predicted effects. Similarly, in a registered replication report, Hoogeveen and colleagues (2018) showed that belief in a controlling god did not increase after a control threat manipulation in comparison to a control affirmation condition, thereby failing to replicate past work on the topic. Other researchers also attempted to replicate the effects of threats to control induced by event recall manipulations on conspiracy theory beliefs without success and a recent meta-analysis found a small and non-significant effect of threatened control on conspiracy beliefs (Stojanov et al., 2020; Stojanov & Halberstadt, 2020). Table 1 presents an overview of selected studies that explicitly focus on the aim of replicating previous findings. Importantly, most of the replication attempts were conceptual, rather than direct replications, as either the type of experimental manipulation or the type of outcome variable differed from the original studies. One exception was the study by Hoogeveen and colleagues (2018). Thus, the question remains: What are the reasons for these replication difficulties?

**Table 1:** Replication attempts of studies using the recall-based manipulations of control

Original research			Replication attempt			Type of replication
Authors	Manipulation	Dependent Variable	Authors	Manipulation	Dependent Variable	
Whitson and Galinsky (2008)	Free recall: High vs. low control	Visual pattern perception in a snowy pictures task	van Elk and Lodder (2018)	Negative event recall: High vs. low control	Illusory contingencies	Conceptual
				Negative event recall: High vs. low control	Illusory contingencies – perceiving non-existing rules	Conceptual
	Free recall: High vs. low control	Illusory Pattern Perception (snowy pictures task)		Reading 64 vignettes about high vs. low control (within-subjects)	Illusory Pattern Perception (snowy pictures task)	Conceptual
	Studies 3, 4, and 6 using recalled-based manipulations to test the illusory pattern perception hypothesis			Negative event recall: High vs. low control (within-participants)	Illusory agent detection	Conceptual
	Free-recall: High vs. low control	Conspiracy perceptions		Negative event recall: High vs. low control	Conspiracy Belief Questionnaire	Conceptual
	Free-recall: High vs. low control	Conspiracy perceptions	Stojanov et al. (2020)	Free recall: High vs. low control	Conspiracy beliefs (Studies 1, 3–6)	Conceptual
Greenaway et al. (2013)	Free recall: High vs. low control	Revised Paranormal Belief Scale (belief in astrology, precognition)	van Elk and Lodder (2018)	Unsolvable Anagram task	Belief in Precognition (e.g., belief that future events can affect us etc.)	Conceptual
Kay et al. (2008)	Positive event recall: High vs. low control	Belief in existence of God as controller	Hoogeveen et al. (2018)	Positive event recall: High vs. low control	Belief in existence of God as controller	Direct

(1) If not stated otherwise, the design of the studies was between participants, (2) All types of replications of the original studies are included in this overview. However, this is not an exhaustive review of the studies that applied recall-based control manipulations

Van Elk and Lodder (2018) argue that the efficacy of experimental autobiographical recall manipulations – in which participants are instructed to recall their personal memory of having-or-not-having a feeling of control – is questionable. Hoogeveen et al. (2018) make a similar argument, suggesting that experimental manipulations of (lack of) control may not be effective in shifting beliefs in God but that individual differences in the experience of control may be more clearly related to religious beliefs. Overall, one might claim that the effects either do not exist or are limited to specific experimental designs, procedures, sample types, etc. Thus, one reason for failed replication attempts might be the much-discussed abuse of experimenter degrees of freedom. However, another important, albeit less-discussed, reason is that we have little understanding about what is being manipulated in these experimental approaches.

Specifically, experimental procedures based on the recall of autobiographical memories (also referred to in literature as *mindset-priming* procedures) gained much attention in the personal control literature (Kay et al., 2008; Rutjens et al., 2013; Whitson & Galinsky, 2008). However, to detect an effect of these types of experimental manipulations of control on any type of variable of interest, one has to assume that: (a) these are valid ways to induce a state of (lacking) control; (b) different versions of such procedures induce a comparable type of control experience and (c) this experience transfers to subsequent measures of perceptions and beliefs. It is plausible that recall-based manipulations of personal control generate a whole range of experiences, with only some relating to personal control. It is also plausible that the varied experimental procedures evoke different aspects of control-related experience (e.g., its emotionality, intensity, etc.).

The failure to validate experimental manipulations of control might limit our ability to replicate research findings. Recently, Chester and Lasko (2021) alerted the field to this problem when they observed that experimental manipulations of psychological constructs might not actually influence their intended mental processes. This might be due to a lack of attention to construct validity of experimental procedures. Here, we wonder if this issue might play a role in undermining the validity and replicability of research on the effects of lacking personal control on social cognition. In this article, we probe the construct validity of manipulations of (lack of) control and discover that while scholars believe they are manipulating one thing, they might in fact be manipulating many things at once, some of which do not include a manipulation of control. Therefore, one may ask whether we really know what type of memories are retrieved in the experimental procedures based on the recall of autobiographical memories. The present study aims to answer this question.

## Construct validity of experimental manipulations of control

A recent analysis of existing experimental manipulations used in social psychology and published in JPSP (348 experimental manipulations obtained from 49 articles) performed by Chester and Lasko (2021) found that 48% of manipulations relied on only face validity. That is, no pilot validity testing, no manipulation checks and no citations were added to justify the effectiveness of the manipulations. Only one-third of manipulations were paired with a manipulation-check measure, and many of these were single-item self-report measures. Additionally, manipulation checks might not assess whether the experimental procedure induced a certain psychological state (e.g., negative emotions, powerlessness, lack of control etc.) but whether the participants correctly recalled an event that involved a given state (e.g. when asking about the amount of experienced emotions / power / control etc. in that recalled situation). If the state was not induced, then the validity of such manipulations is questionable. In fact, proper manipulation checks can be highly useful for validity control and a catalyst for improving the quality of psychological science (Fiedler et al., 2021). Threats to a manipulation’s construct validity can also be related to confounding aspects of the experimental procedure that are not related to the manipulation itself (e.g., an inappropriate control condition; see also the discussion on so-called “instrumental incidentals”, Chester & Lasko, 2021). For example, the control condition might differ from the experimental one not only in the amount of experienced control but also in the domain of exercised control – personal or interpersonal, which might inadvertently impact the outcome variable. Another important threat to construct validity is a lack of specificity of the manipulation, meaning that the manipulation might have an impact on a broad range of constructs and not specifically on the target construct. For example, manipulating lack of control can not only lower feelings of personal control, but also increase negative emotions, lower self-esteem, increase anxiety and induce a generalized state of uncertainty (e.g., see Bukowski et al., 2019; Sedek & Kofta, 1990; Schneider, 2022).

In line with this reasoning, we focus on three types of factors that affect the construct validity of widely used experimental manipulations of personal control. Firstly, we identify the nomological network related to the construct of personal control. We do this by analyzing the main aspects of experiences related to a sense of (lacking) control, for example, its emotionality, duration, relevance, perceived importance, etc. Secondly, we examine how manipulations of control impact these aspects of experience to check whether there are confounding aspects of the manipulation that lead to different types of experiences when inducing low vs. high control. Thirdly, we analyze to what extent different aspects of control related experiences vary between the most commonly used versions of experimental procedures. That is, we explore how diminished vs. enhanced control evoked by different versions of recall-based control manipulation procedures is phenomenologically experienced.

## Recall-based manipulations of personal control

When studying the consequences of feelings of control (and lack thereof) on social cognitive processes, previous studies have mostly relied on procedures based on the recall of autobiographical memories (e.g., “recall an event from your personal past when you did (or did not) have a feeling of control”). Such priming procedures have commonly been applied to activate various types of mental constructs, from knowledge structures to goals, from intentions to mindsets (Bargh & Chartrand, 2000; Forster & Liberman, 2007). The use of such methods is widespread across a variety of research domains in psychology, such as self and identity, social power, and status (Galinsky et al., 2003; Karyłowski & Mroziński, 2017; Keltner et al., 2003). However, similar to the research on personal control, the results obtained in experiments using recall-based methods (often referred to in the literature as mindset or episodic priming procedures) have not been consistent and conclusive (Ebersole et al., 2016; Heller & Ullrich, 2017; Imada et al., 2023).

A common assumption when applying such recall-based experimental methods is that a similar psychological state (e.g., of low personal control, power, etc.) is made cognitively accessible, even if there is some variation due to variability in personal experiences. This assumption, however, might not be fully warranted given what we know about autobiographical memory. In particular, there are various types of memory-retrieval effects that may be triggered by different memory-based procedures. Importantly, they may lead to different memories that can influence the effectiveness of these procedures and their suitability for experimental research.

For example, theories of autobiographical memory distinguish between effortless vs. effortful retrieval (Conway, 1996; Conway & Pleydell-Pearce, 2000). An important result emerging from this literature is that the subjective amount of effort experienced when retrieving memories might alter the phenomenological characteristics of memories. For example, compared to effortful memories, spontaneously retrieved memories tend to be more vivid, clear, personally significant, field perspective oriented, and emotional (Barzykowski & Staugaard, 2016; Harris et al., 2015). Yet, it is unknown to what extent different versions of recall-based control manipulation procedures involve high or low amounts of effort. Overall, it might be that certain types of memory-based procedures that require a more laborious recall process can activate memories that vary in their accessibility levels (i.e., memories that are less vivid, effortful, self-oriented, etc.), but no study has systematically examined this.

Another methodological issue that renders the use of recall-based procedures problematic is related to the recent findings showing that the variance on the individual level of analysis is up to four times larger than the variance at the group (aggregated) level (Fisher et al., 2018). This might be because most psychological processes have an individually variable and time-sensitive nature. We think that this high variability of the intraindividual level may be even more likely for experimental procedures that rely on individual memories and experiences. For instance, it may be that the recall of autobiographical memory procedure for different individuals actually taps into different aspects of personal control but also that it engages specific memory-retrieval processes to a different degree (e.g., effortful vs. effortless). We wonder if when manipulating personal control by applying such procedures, whether the result is a variable experience that does not consistently activate the construct of interest. Thus, it seems important to focus on the types of contents the participants produce when recalling instances of (lack of) control, to understand what constructs of control and perhaps also other, not related to control, get activated using specific memory-based experimental procedures.

## Overview

In this article, we provide a critical view of current practices in experimental research on the social psychology of personal control. Specifically, we focus on the use of experimental procedures based on the recall of autobiographic memories. The aim of Study 1a and 1b was to examine what aspects of the personal control experience are evoked by two types of commonly used recall-based manipulations of personal control – one developed and applied by Whitson and Galinsky (2008) and one developed by Kay et al. (2008). We also examined the extent to which feelings of control were evoked in low and high control induction conditions. Studies 1a and 1b consisted of a qualitative re-analysis of existing datasets, in which two types of recall-based manipulations were used.

In Study 2, we further examined how different recall-based manipulations shape participants’ phenomenological experiences but this time experimentally inducing memories related to control or lack of control. As in Study 1b, we included the free recall (Whitson & Galinsky, 2008), and positive event recall procedure (Kay et al., 2008), but additionally we introduced the negative event recall procedure (Rutjens et al., 2010). This way, by focusing on the recall of positive or negative events, we could also examine role of the emotionality in the existing recall-based control manipulations. We tested for the same type of comparisons as in Study 1a and 1b – between low and high control conditions and between different versions of manipulations but additionally assessed different aspects of personal control. Overall, Study 2 provided a pre-registered, experimental test for our predictions regarding a differential impact of three different control manipulations on the same set of outcome variables.

## Study 1a

### Data source and participants

We re-analyzed data from four studies on the impact of control experiences on social norm following intentions, using the free recall procedure developed by Whitson & Galinsky (2008). We had access to 662 answers provided by participants.^1^ A judge first read all the answers and classified them as either a valid recall or not (i.e., related to the instruction in a meaningful way – we excluded participants who answered that they do not have such a memory and those who gave irrelevant answers, i.e., containing one word). This resulted in a final sample of 627 undergraduate student participants (480 female, 147 male, age, *M* = 20.34, *SD* = 1.85).

### Procedure

Participants were randomly assigned to either the high or low control condition. They read the following instructions: “Please recall a particular incident in which something happened, and you did not have control over [you were in control of] the situation. Please describe the situation in which you felt a lack of control [felt in control] – what happened, how you felt, etc.”. We deleted the words “any” [control] and “complete” [control] used in the original manipulation due to a very high number of participants in a previous study who declared it is impossible to feel complete control or complete lack of control in life.

### Content analysis

Memories were then coded by two independent judges according to the following categories: (1) Specificity: specific or general, (2), Valence: positive or negative, (3) Goal completion: success or failure oriented, (4) Type of ending: happy or sad ending of the story, (5) Context: educational or social^2^, (6) Coping style: active or passive, (7) Reference to emotions: with or without reference to one own’s emotion and feelings, (8) Level of control: high or low control, (9) Control domain: physical or cognitive, (10) Self- vs. other-focus: Me or other oriented (lack of) control, (11) Sphere of control: intrapersonal or interpersonal, (12) Duration: brief (e.g., relating to a particular situation/time) or long-lasting (lasting over an extended time period) (lack of) control, (13) Source of control: external vs. internal. Thus, we were able to codify the main aspects of memory (dimensions 1–4), the main memory theme and/or content (dimensions 5–7) and the main domain/type of control/the lack of control that memories referred to (domains 8–13). Importantly, judges were sufficiently trained beforehand to ensure good understanding and proficiency in coding. To this end judges were: (1) provided with coding definitions and manuals (see Table 1, Supplementary Materials: https://osf.io/mk7s2/?view_only=c09801257465477493d7364887d030ee) which, to ensure good understanding of the categories, were discussed and any doubts were solved prior data collecting phase; (2) asked to code small number of entries, which was followed by discussion about the categories and any misunderstanding and/or doubts about the coding system were solved by the discussion; (3) asked to code the all remaining entries once they declared full understanding of how to use the codes; (4) asked to thoroughly discuss the disagreements to find the final agreed category, however, if the agreement was not easily found, the memory was deemed as undecidable and fell into the third category ‘other’.

For example, memories describing being in charge and/or having control over something were coded as high control memories (e.g., *I was very pleased because I controlled the whole situation*), while memories describing a lack of power/control and/or helplessness were coded as low control (e.g., *I could not do anything, this was totally beyond my control*). In line with the literature on autobiographical memories (e.g., Williams, 1996; Barzykowski & Staugaard, 2016, 2018; Barzykowski et al., 2019), memories were coded as specific, one-off memories of events that happened at a particular place and time (e.g., the day I met my partner) or general memories that relate to repetitive events or events that lasted over an extended time period (e.g., *vacation in Spain*). Next, in line with the study by Walls et al. (2001; Barzykowski et al. 2019), memories focused on learning or subject matter were coded as educationally oriented (e.g., *doing schoolwork*), while memories focused on interacting or socializing with other people were coded as socially oriented (e.g., *dancing with a partner*). Memories describing being active (e.g., *doing something to change the situation*) or being passive (e.g., *being helpless, not taking any action*) were coded as active or passive, respectively. Memories relating to physical (e.g., body, health/sickness, doing something manually, memory relating to feelings and/or emotions) or cognitive aspects (e.g., mind, thoughts, cognitive processing, understanding) were coded as either physically or cognitively oriented. Memories relating to one’s individual life (e.g., private matters, own decisions, the context of personal life, behaviors) were coded as self-oriented, while descriptions relating to other people (e.g., friends, spouse) were judged as other-oriented. Next, memories describing having control or lack of control over, for example, one’s own life, activity, or development were coded as intrapersonal, while those relating to relationships or social interactions were judged as interpersonal. Finally, memories describing the source of the control situation as random and external (i.e., beyond one’s control, e.g., being sick) were coded as externally control based, while situations relating to one’s (cap)abilities, efficiency, effort (e.g., studying hard) were coded as internally control based.

Examples of memories identified by independent judges as “other” were unrelated to the given category. Such entries accounted, on average, for: .5% (specific or general memory), 5.1% (positive or negative memory), 26.5% (success or failure oriented memory), 30.1% (happy or sad ending memory), 37% (educationally or socially oriented), 2.1% (active or passive memory), .2% (with or without emotion reference), .3% (high or low control), 1.8% (physical or cognitive control), .5% (Me or other control), 1% (intrapersonal or interpersonal control), .5% (short or long lasting control), 3.2% (external or internal source of control) of all memories recorded by participants. Importantly, as all disagreements were resolved ﻿by the discussion, ﻿we feel confident that the categories were reliably evaluated.

### Results

The inter-rater reliability was high, with a Cohen’s κ coefficient ranging between .37 (for short or long-lasting lack of/control) and .95 (for high/low control), with an average κ score of .59. Importantly, Landis and Koch (1977) considered a Cohen’s κ of .41 and .60 to be “moderate agreement”, a κ between .61 and .80 to be “substantial agreement”, and a κ above .81 to be “almost perfect or perfect agreement”. Thus, it can be argued that while the lowest Cohen’s κ (i.e., .37) was not high but still acceptable agreement, the rest of Cohen’s κ were at least .40 and thus acceptable. Therefore, the given categories were valid and reliable. Table 2 in Supplementary Materials provides all κ's for each category.

**Table 2:**
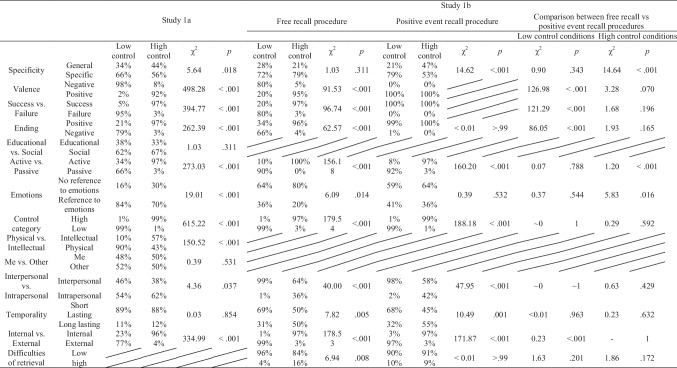
Overall mean ratings for coded categories, percentages of memories as a function of experimental condition in Study 1a and 1b

We conducted a χ^2^ test to check for differences in the content of memories between the low and high control condition. We observed that in the low (vs. high) control condition the locus of control was more external (vs. internal) and the memories were more negative in valence, more specific and often included an episode of failure rather than success. In the high control condition, 97% of participants described the story as having a positive vs. negative ending. Interestingly, in the low control condition 21% of memories contained positive endings as well, which may suggest that participants described situations that contained an experience of control restoration. Additionally, in the low (vs. high) control condition memories of participants included more references to emotions. In the high control condition, 97% of the stories were coded as active, but in the low control condition 34% of the entries were coded as active (vs. passive). This means that every third participant in the low control condition described his or her attempts to control the situation. Additionally, in the high control condition, participants were more likely to describe intrapersonal (vs. interpersonal) and more intellectual (than physical) experiences. However, this finding could be contingent on the specific sample of students. All the comparisons between low and high control conditions are depicted in Table 2 below.

In sum, this study revealed substantial variability between experimental conditions that were designed to induce experiences of low and high personal control. In fact, the recalled memories differed not only in the amount of experienced control and locus of control, but also in other aspects, such as valence, reference to emotions and a sense of failure or success at the end of the recalled event. This might indicate that this type of procedure activates other psychological constructs apart from control, like negative emotions related to personal failure. This finding is critical because it shows that different control manipulations are not equivalent in terms of the psychological processes induced (e.g., control vs. negative emotionality) and therefore they can have also differential impact on the same outcome variables (e.g., enhancing negative social biases only when the aspect of negativity is salient). Additionally, we identified various aspects of the control construct itself that differ between low and high control conditions, such as the domain in which control was exercised (physical vs. intellectual) and the sphere of control (intra- vs. interpersonal). Finally, the content analysis also revealed variability in the type of responses to lack of control experiences – 34% approached the lack of control inducing situation in an active way and 21% managed to restore control at the end. These results cast doubt on the extent to which a low control condition specifically induced a state of uncontrollability/helplessness or just evoked a series of memories that were generally more negative and involved feelings of personal failure.

## Study 1b

Since Study 1a revealed the importance of emotionality and valence of the recalled episodes as a potentially confounding aspect of the recall-based control manipulation, in this study we compared the contents described in the free recall (as in Study 1a) and additionally positive events recall procedures (Whitson & Galinsky, 2008; Kay et al., 2008).

### Method

#### Data source and participants

We used data from a study that compared the effects of two different memory recall tasks that manipulated control salience on the approval of social hierarchies. This study was conducted by the Department of Social Psychology at the University of Leipzig (Lautenbacher & Fritsche, 2023; Study 2), for which 454 participants were recruited via diverse social media platforms (gender: 303 women, 128 men, 20 non-binary, three with no answer; age: below 25 years: 244, between 25 and 34 years: 155, between 35 and 44 years: 33, between 45 and 54 years: 17, between 55 and 64 years: 3).

#### Procedure and materials

Upon agreeing to participate in the study, participants completed one of two memory recall tasks to manipulate salience of control (Kay et al., 2008; Whitson & Galinsky, 2008). Half of the participants were randomly assigned to a free recall control manipulation. In this manipulation, participants were asked to write about a recent experience over which they either had full or no control, without specifying what valence that experience should have (“Please try and think of something that happened to you in the past few months that was [not] your fault (i.e., that you had [absolutely no] control over). Please describe that event in no more than 100 words.”). We adjusted the wording of the original manipulation to make it more comparable with the one used by Kay at el. (2008). The other half was randomly assigned to a control manipulation used by Kay et al. (2008), which was very similar to the free recall manipulation, apart from the fact that it attempted to keep the valences of the described situations constant. Precisely, we asked participants to think about a *positive* experience of the recent past in which they either had full or no control (“Please try and think of something *positive* that happened to you in the past few months that was [not] your fault (i.e., that you had [absolutely no] control over). Please describe that event in no more than 100 words.”).^3^ Subsequently, participants completed further tasks and measures not related to the purpose of this analysis (Lautenbacher & Fritsche, 2023).

### Results

Two independent judges coded the memories of all participants. They were not privy to hypotheses and research questions of the present study. We only provided them with the coding guidelines for the categories described in Study 1a and asked them to conduct a deductive content analysis. As a first step, the research assistants only coded the memories of the first 100 participants to ensure that sufficient interrater-reliability was reached. For the coding of the first 100 participants, the two coders reached a substantial agreement across all categories of *κ* = .66. As in Study 1a, all disagreements were subsequently resolved in a discussion between both raters. Then, the research assistants continued with coding the memories of all remaining participants. A substantial interrater-reliability was found across all categories after all memories were coded (*κ* = .65; see Table 2 in Supplementary Materials for agreements on each category). Again, the remaining disagreements were resolved in a discussion between both raters resulting in the final coding dataset used in the analyses. For simplicity reasons, we reduced the overall number of categories and added one, namely difficulty of retrieval (e.g., How difficult was it to recall the event?), which has been identified as an important variable that influences the reliability of memory-based power manipulations (Lammers et al., 2017).

The results obtained for the free recall control manipulation indicated that high and low control conditions differed in each category apart from specificity. The analyses replicated most of the findings from Studies 1a; Participants in the Low (vs. High) control condition described more memories that were negative (vs. positive) and made greater reference to emotions. Low control episodes included aspects of failure rather than success and the agent was passive (vs. active). Even though high control condition participants were more likely to describe situations with a positive, “happy” ending, 34% of low control condition participants still described stories that ended positively. Again, in the low (vs. high) control condition the memories had more interpersonal (vs. intrapersonal) aspects. Unlike in Study 1a, in the low (vs. high) control condition participants described briefer, rather than long-lasting situations. Source of control was mostly external in the low control condition, and internal in the high control condition. Finally, in the low (vs. high) control condition participants were also more likely to mention difficulties with retrieval. Table 2 presents a summary of the comparison between high and low control conditions within each manipulation.

Next, the same coding procedure was conducted for memories recalled in the positive event recall control manipulation. Consistent with the assumptions of this type of manipulation, only events with positive valence were recalled in the low and high control conditions. The conditions did not differ significantly in the amount of reference to emotions; in fact, emotional states were not referenced in most of the recalled events. As in Study 1a, most participants described an experience of being passive in the low control condition versus being active in the high control condition. In the low (vs. high) control condition participants tended to describe more interpersonal memories. While in the low control condition, most memories applied to brief situations, in the high control condition most were related to long-lasting situations. In the low control condition, the source of control was mostly categorized as external, and in the high control condition – as internal.

Next, we conducted a series of comparisons between free recall vs. positive event recall manipulations. First, we compared both manipulations in the low control and high control condition. For a summary, see Table 2. We observed three differences between both manipulations in the low control condition. First, due to the nature of the manipulations, all positive event recall memories were positive, whereas only 20% of them were positive for the free recall manipulation. Similarly, while all the memories in the positive event recall manipulation described success, in the low control condition of the free recall manipulation, only 20% of entries described success. Finally, while in the positive event recall manipulation the vast majority of the stories had a positive, “happy” ending, in the free recall procedure the majority of memories had negative, “sad” endings. However, it is still worth noting that 34% of the stories in the free recall manipulation were classified as having a positive ending.

We observed differences between the manipulations in the high control condition. First, in the free (vs. positive) recall manipulation participants described more specific situations of having control. Second, while in the free recall manipulation all participants described experiences of being active, in the positive event recall manipulation some of them were coded as passive. Finally, in the free (vs. positive) recall manipulation few participants referred to emotions. No other comparisons were significant.

### Discussion

The results from Study 1b revealed a very similar pattern for content analyses between low and high control conditions to Study 1a. That is, the recalled memories related to the low vs. high control differed in their valence, reference to emotions, feelings of failure, and active coping intentions. Study 1b allowed us to compare two types of manipulations that differed in the valence of the recalled events. By focusing on only positive aspects of control, the Kay control manipulation managed to resolve the potential confound between control and a mixed emotional valence of the recalled episodes. In this sense it seems to be an improvement with regard to its ability to assess control and not merely negative emotionality. However, it differed in terms of the construct of control that was measured. Specifically, a vast majority of memories recalled in the low control conditions in the free recall manipulation were related to examples of personal failure and ended badly, whereas in the positive events recall manipulations the memories were related to success and the situation ended positively. When analyzing the specific contents of the stories one can easily notice that the lack of control experience described in the positive events recall manipulation is related to a sense of randomness or chance that turns out to be beneficial for the acting person. Thus, there is minimal self-agency involved, with memories focusing on the unpredictability of the environment, whereas the memories recalled in the free recall procedure are related to the efficacy of one’s personal actions.

The results from Study 1a and 1b suggest low comparability of the low and high control experimental conditions, especially when using the free recall manipulation. In other words, when using this procedure, people not only feel more (or less) in control, but also feel a host of other things. One implication is that when scholars use this manipulation and find an effect, it will be unclear as to why the effect comes about – is it about lack of control or about being in a bad mood or about having to exert effort?

In the next step, we aimed at comparing three different procedures, two of which were specifically designed to experimentally control the valence activated by the recall procedure by directly asking participants to either recall only positive or negative memories related to feelings of control. One potential drawback of studies 1a and 1b was also an unbalanced sample in terms of participants’ gender (more women than men). As this might be an important factor that affects their responses to control manipulations, we sought to have a more balanced sample in Study 2.

## Study 2

In the previous two studies we identified key aspects related to the experience of personal control that are affected by two commonly used recall-based control manipulations. In this study, we focused mainly on the aspect of emotional valence of the recalled event and compared three different control manipulation procedures widely used in the literature. Specifically, we applied two of the procedures used in the previous studies: One that asks participants to recall freely memories of (lacking) control (Whitson & Galinsky, 2008), one that instructs them to recall only positive (un)controllable events (Kay et al., 2008) and one that directs the participants to remember negative (un)controllable events only (Rutjens et al., 2010). The main question is whether different recall-based control manipulations affect specific phenomenological properties of the control experience to a different degree, and whether these are related to the emotionality of the recalled event, as well as its length, intensity, and the difficulty of retrieval^4^.

### Participants

A total of 641 participants completed the whole survey. The participants were recruited via an online research company in Poland (Pollster: https://pollster.pl) and the survey was conducted in Polish. We excluded 17 participants who failed to complete the attention check properly and 118 participants who could not recall any event. This left us with a final sample of 506 participants (291 women, 215 men, *M* = 39.61, *SD* = 10.88). We told participants that they would take part in a study on how people remember their personal past. This research was approved by the appropriate institutional ethics committee, participants provided their written consent before participating in the study.

### Procedure

We present the following measures in the order of their appearance in the experimental procedure.

**Mood: Time 1.** At the beginning we measured participants’ mood on a scale from 1 (very bad) to 7 (very good).

**Control manipulation.** Next, participants were randomly assigned to one of seven conditions: free recall (high vs. low control; Whitson & Galinsky, 2008), recall of positive events (high vs. low control; Kay et al., 2008), recall of negative events (high vs. low control; Rutjens et al., 2010) or a baseline control^5^. Full wording of the manipulations was as follows:

***Free recall*** (Whitson & Galinsky, 2008)*.* “Please recall a particular incident in which something happened, and you did not have any control over [you were in complete control of] the situation. Please describe the situation in which you felt a complete lack of control [felt in complete control] – what happened, how you felt, etc.”

***Negative events recall*** (Rutjens et al., 2010). “Research has shown that people remember positive and negative events in different ways. In this research, we are interested in your recollection of a specific event. Please try to think back to an unpleasant event or situation that you experienced not too long ago, over which you had absolutely no control [total control]. Can you remember such a situation or event? Try to describe this uncontrollable event. “What happened and how did you feel?”. After answering these questions, participants in the low control condition read the following part of the task: “Next, we would like you to think about the fact that it is very difficult to predict what will happen to you tomorrow, next week, or in a year from now. There are many uncontrollable factors that determine your fate and the events that have yet to take place. Life is simply unpredictable – you can never know what the future will look like. Please think about this for a bit and then try to name three arguments in favor of the fact that the future indeed is uncontrollable and unpredictable”. Participants in the high control condition read different instruction: “Next, we would like you to think about the fact that you are in control over your own life. Although there are many things that can cross your path in life, you are able to make important decisions, take things into consideration, avoid certain situations and approach others. You are in charge of your own future. Please think about this for a bit and then try to name three arguments in favor of the fact that you have control over your own future”.

***Positive events recall*** (Kay et al., 2008). **“**Please try and think of something positive that happened to you in the past few months that was [not] your fault (i.e., that you had [absolutely no] control over). Please describe that event.”^6^

***Baseline control.*** “Please recall the last time you walked a familiar route, e.g., from home to work, to the university or to the store. Please describe this situation”.^7^

**Task completion check**. Next, we asked participants whether they had described the situation in the previous task, to avoid that participants who did not recall any memory answer further questions. Participants who said “no” could not proceed with the study.

**Mood: Time 2.** Afterwards participants answered the same question about mood again.

**Feelings questionnaire.** We used the SPANE questionnaire (Diener et al., 2010), which contained 12 items to assess positive and negative feelings (six items for each type of feelings).

**Perceived personal control**. A generalized perception of personal control in life was measured with three items (Greenaway et al., 2015); “I have control over my own life", “I can live the way I want to live”, “My life is determined by my own actions”, α = .85.

**Manipulation checks**. We used four items to measure how participants experienced the event at the time it happened in terms of controllability: “To what extent did you feel you were in control when the event happened?”, “To what extent do you assess that you were in control of this situation?”, “To what extent did you feel helpless when the event happened?”, and “How unpleasant was this situation then?”. Participants answered on a scale from 1 (not at all) to 7 (very much). A factor analysis revealed that the last item did not load on the same factor as the three first ones. Thus, we calculated an index of perceived uncontrollability over the recalled event from the first three items that directly referred to the notion of control, α = .87.

**Sense of control.** We first asked participants whether they tried to influence the recalled situation in any way. Subsequently, to investigate different aspects of personal control proposed by Skinner (1996), we asked them the following questions: “To what extent do you feel that you knew what to do in the described situation to achieve the desired effect?”. “To what extent are you sure, that this behavior led to expected results?” (action strategies and efficacy beliefs), “To what extent do you feel that you had the opportunity to achieve the desired effect?”, “To what extent do you feel that you had the appropriate competences to try to achieve the expected results?” (competences and ability beliefs), “To what extent do you feel that you were in control of the situation?”, “To what extent do you feel that you achieved the expected results in the described situation?” (sense of personal control). Participants answered on a scale from 1 (not at all) to 7 (very much). We performed a factor analysis to check whether a three-factor solution emerged but as we only found a coherent one factor solution, we calculated one index for this scale labeled as sense of control, α = .96.

**Fatigue.** We measured task-related fatigue with nine items based on Sedek and Kofta (1990); “I felt discouraged when recalling and describing the event from my own past”, “My mind was running blank when recalling and describing the event from my own past”, “I was fully involved in recalling and describing the event”, “I found it very hard to think”, “It was difficult for me to get motivated to recall and describe the event”, “I was distracted and could not focus”, “Recalling and describing the event was a pleasant activity”, “The task of recalling and describing the event was very interesting”, “Very few ideas of a specific event came to my mind”. Items 3, 7 and 8 were reversed coded, α = .80.

**Importance**. We asked participants how important it was for them to have control in the described situation. They answered on a scale from 1 (not at all important) to 7 (very important).

**Difficulty of retrieval.** We measured ease of retrieval with three items (Lammers et al., 2017); “How difficult was it to recall the described memory related to control?", “How difficult was it to recall the details of this event?”, “How difficult was it to recall how you felt during the event?”, α = .86.

**Phenomenological characteristics of the event memory**. First, we asked participants a yes/no question on whether they tried to be active in the recalled situation and whether they tried to influence its result. Those who indicated “yes” answered two additional questions: one about the difficulty of the situation - “How difficult was the situation for you when it was happening?” (1 – not at all difficult, 7 – very difficult) and the other about the effort invested in impacting the event – “How much effort did you have to invest to influence course of events in this situation?” (1 – not at all, 7 – very much). Next, we asked eight questions about the quality of the given memory, such as: “How well do you remember this memory?”, “How detailed is this memory?”, “How vivid and clear is this memory?”, “How pleasant this memory is for you?”, “How intense are the emotions related to this memory?”, “How important is this memory for you?”, “How special was this memory?”, “How tired are you due to recalling this memory?”. We performed on all these items a principal component factor analysis and discovered a three-factor solution: Factor 1 was related to effortfulness of coping (i.e., How hard and difficult was the recalled situation, how tired the recall made them feel, and how (un)pleasant was the memory of the recalled situation). Factor 2 was related to the clarity of recalled memories (their quality, detail, vividness of memories), and Factor 3, labeled as memory distinctiveness, referred to how important, special, and intensive the recalled events were). Based on this analysis, we created an index of effortfulness of coping (Factor 1; α = .86), an index of clarity of the memory (Factor 2; α = .85) and an index of distinctiveness of distinctiveness of the memory (Factor 3; α = .72).

**Self-mastery**. We used the seven-item self-mastery measure developed by Pearlin and Schooler (1978), α = .88.

**Demographics.** We measured age, gender, job status, education, and nationality.

**Debriefing.** At the end we debriefed participants and recommended a short film on how to cope with negative emotions.

The study was pre-registered. 8

### Results

We focused on five categories of variables: (1) Manipulation checks of perceived control related to the recalled event, (2) General perceptions of control, (3) Event-related emotions, (4) Effortfulness of the recall process, and (5) Phenomenological characteristics of the event memory. We describe the results referring to these categories.

Next, we applied a 2 x 3 ANOVA design, which allowed us to compare participants’ responses to each type of experimental manipulation comparing the differences between the low (vs. high) control conditions but also between the three different types of control manipulations (free recall, positive events and negative events recall). Additionally, we compared the results for high and low control conditions with the baseline condition using a series of *t* tests.^9^ All analyses are summarized in Table 3.

**Table 3:**
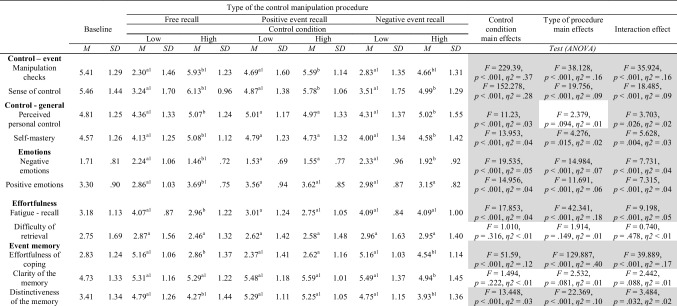
Mean ratings and standard deviations for Study 2 as a function of the type of the control manipulation procedure and level of control

Means with different literal subscripts (e.g., a, b) are significantly different from each other. Means with the numerical subscript 1 are significantly different from the baseline

### Manipulation checks

#### Perceived control over the event

First, we checked whether participants in the low (vs. high) control condition experienced less control related to the recalled event in all three procedures. This is a typical memory check that is often applied as a manipulation check in these types of procedures. All the differences between conditions in the three types of control manipulation were significant. Additionally, there was a significant interaction effect between control level and type of manipulation, indicating that perceived control over the event was lower in the free and negative recall compared to the positive event recall condition, in which the value in the high control condition was comparable to the baseline level (i.e., showing only a relative drop of control in the low control condition).

#### Sense of control

We also compared high and low control conditions using the event-related sense of control measure and found significant differences between high and low control conditions for all three manipulation types. Additionally, high control conditions in the positive and negative recall manipulations did not differ significantly from the baseline (see Table 3).

### Personal control

Next, we checked whether the effects of the manipulation transferred onto two measures of general personal control over life: perceived personal control (Greenaway et al., 2015) and self-mastery (Pearlin & Schooler, 1978). We found that participants who were asked to recall specific uncontrollable events from their lives subsequently felt significantly less control over their lives overall after the free recall and the negative events recall manipulations, but not in the positive events recall manipulation (high vs. low control comparisons). There was also a significant interaction effect, partly driven by an elevated perception of personal control in both conditions of the positive event recall procedure. Additionally, for the positive events recall procedure the values of perceived personal control in both high and low control conditions did not differ significantly from the baseline, whereas in the free recall and negative events recall manipulations only the high control condition did not differ from the baseline.

### Emotions

#### Mood – pre- post-manipulation measure

We performed a repeated measures ANOVA to see whether the manipulation affected participants’ mood. We used reported mood before and after the manipulation as within-subjects factor and type of control manipulation procedure (free recall, positive and negative event recall) and level of control (high vs. low) as between-subjects factor. We did not observe a main effect of mood (*F* < 1) but we did find a significant interaction between mood, level of control and type of procedure (*F*(2, 389) = 6.636, *p* = .001, *η*^*2*^ = .03). Interestingly, only in the lack of control condition of the free recall procedure did participants report decreased mood in the second measurement of mood, indicating that the manipulation significantly decreased their mood state (*p* < .001, *d* = 0.49).

#### Negative emotions

Participants experienced higher levels of negative emotions in the low (vs. high) control conditions after the free recall and the negative events recall manipulations, whereas there was no significant difference in negative emotions experienced in the positive events recall manipulation. Also, the level of negative emotions did not differ between both, positive recall manipulation conditions and the baseline. In the negative events recall manipulation only the high control condition did not differ from the baseline.

#### Positive emotions

After the free recall manipulation, participants experienced less positive emotions in the low (vs. high) control. In the positive and negative events manipulation, the difference between high and low control was non-significant. Also, the low control condition in the positive events recall manipulation and the high control condition for the negative events recall condition did not differ significantly from the baseline. Additionally for both types of emotions we found interaction effects, mainly driven by the large disparities of responses in the free recall procedure.

### Effortfulness of the recall process

The next group of variables assessed the effort needed to recall a specific memory of a control-involving event but also the effort needed to cope with the described situation.^10^

#### Fatigue during recall

Participants in the free recall condition differed significantly in the level of experienced fatigue with the recall process between high and low conditions, showing more fatigue in the low (vs. high) control condition. We did not observe such differences in the positive and negative recall conditions. Interestingly, we found an interaction effect showing that the negative event recall procedure was overall more exhausting than the positive event recall one and comparable to the low control condition in the free recall procedure. Also, both conditions in the negative event recall procedure generated significantly higher scores on the fatigue measure as the baseline condition. We observed similar results for the effortfulness of coping variable, indicating that feeling tired and fatigued by thinking about the negative events might be a characteristic feature of this type of control manipulation.

#### Difficulty of retrieval

The levels of recall difficulty were comparable across conditions (non-significant interaction and main effects of the manipulations).

### Phenomenological characteristics of the event memory

The last group of variables contained various characteristics of the recalled event memory, such as recalled effortfulness of coping when the event happened, clarity of the memory, and distinctiveness of the memory. Effortfulness of coping was higher in the low (vs. high) control conditions for the free recall and negative events recall procedures, but there were no differences for the positive event recall one. For the memory clarity measure, we found that it was higher in the low (vs. high) control condition but only for the negative event recall procedure.^11^ The results for measures of distinctiveness of the memory revealed the same pattern as for effortfulness of coping, i.e., in the free recall and negative event recall procedures, low (vs. high) control was associated with higher levels of distinctiveness. All of the scores in the low control condition differed significantly from the baseline. However, in the high control condition scores for effortfulness of coping did not differ from the baseline scores for the free recall and positive events recall procedures but were significantly higher than in the baseline in the negative event recall procedure. This result indicates that the high control condition in the negative event recall procedure (unlike the other procedures) activated memories of situations that were difficult to cope with.

### Discussion

The aim of this experimental study was to compare three types of recall-based control manipulations with reference to such categories as perceived control over the event, generalized personal control, emotions, effortfulness and other phenomenological aspects of the memory. We found that all three manipulations have a predicted, positive impact on the applied manipulation checks, that is, participants declared that during the recalled event they felt less control when asked to think of an uncontrollable (vs. controllable) situation. This result seems to support the validity of all three manipulations. However, the appropriateness of this (commonly used) manipulation check is questionable since it measures not perceived personal control after recalling the event, but the amount of control that participants felt when the situation had happened. Therefore, we also measured a generalized perception of personal control after the applied manipulations and found that the free recall and negative events recall manipulations generated stronger feelings of general (chronic) lack of control in life, but that the positive event recall procedure was unsuccessful in inducing a generalized perception of uncontrollability. Additionally, for the positive events recall procedure the level of perceived personal control did not differ between the low control and baseline condition. These results potentially question the validity of the positive events recall procedure as a tool to induce a sense of personal lack of control. These findings show that it is important to make a distinction between control felt in a particular situation and felt after recalling the event as it indicates whether indeed a sense of control or just the memory of control was manipulated. Future studies should address this issue in more detail, ideally measuring both aspects of control it in a within-subject (pre-post-measurement) design. This would allow us to determine with more certainty, which manipulations indeed activate feelings of lacking control or just memories of situations that illustrate lack of control but not necessarily induce the experience of it.

When we analyzed the emotions induced by the different types of manipulations, we found that for negative emotions, only the positive event recall procedure generated comparable levels of negative emotions in both (high and low control) conditions. We also found a comparable level of positive emotions for both; the positive and negative events recall procedures. These results indicate that the positive recall procedure successfully controls for emotional states related to uncontrollability, whereas the free recall procedure is especially confounded by emotion with a strong involvement of negative emotions in the low control condition and a stronger induction of positive emotions in the high control condition.

We next examined effortfulness or fatigue related to the recall process. We found that the free recall procedure differed strongly between conditions in terms of experienced fatigue (the low vs. high control condition generated more feelings of mental fatigue). However, the negative events recall procedure generated a similar level of fatigue in the low and high control conditions, indicating that perhaps negative affective states are related to the experienced effortfulness of thinking about these events. Such an interpretation would be supported by the result of high coping effortfulness during the recall of negative events in low and high control conditions.

Apart from effortfulness of coping, we compared the three types of control manipulation referring to such phenomenological characteristics of the event memory as clarity and distinctiveness of the memories. We found that the positive event recall procedure yielded comparable levels of memory clarity in the high and low control conditions for the free recall and positive events recall procedures but not for the negative events recall one (here the clarity of memories in the high control group was relatively lower comparing to the low control). The distinctiveness of memories was higher in low (vs. high) control conditions for the free recall procedure and negative event recall procedures but not for the positive events one. Interestingly, the negative event recall procedure differed significantly in all dimensions of the memory characteristics between conditions, revealing its weakness in terms of comparability of the two experimental conditions.

Summing up, each control manipulation had different strengths and weaknesses in terms of their validity as a tool to induce experiences of (lack of) control. The free recall procedure heavily relies on emotional states (negative in the low control and positive in the high control condition), which by itself is not surprising but the intensity of experienced negative emotions might vary between participants and between populations. Thus, this manipulation generates considerable variability in emotional experiences, which might itself create potential confounds. The positive event manipulation deals well with this issue, but it does not affect perceptions of personal control, which calls into question the assumption that it manipulates personal control and not a different, although nomologically related construct (e.g., uncertainty). The negative event manipulation generates strong (negative) feelings of uncontrollability but the procedure is overall quite demanding for participants. In particular, the high control negative events condition generates high feelings of fatigue. Also, there was a relatively high selective dropout in the high control condition for this type of manipulation. It seems plausible that participants who decided to quit the study or not to write anything in this experimental condition found it too hard to come up with any example of high control with negative valence. If this would be the case, then such attrition could pose a threat on the internal validity of this procedure and the results obtained with it. Additionally, memories differ in all the measured phenomenological aspects between high and low control, which might indicate that the retrieval process also differs in terms of its quality.

## General discussion

The main aim of this article was to examine the construct validity of experimental manipulations of control (or lack thereof). We focused on a specific type of control manipulation procedure based on recall from autobiographical memory. The assumption behind the use of these procedures is that by reflecting on an event or situation involving personal control, the accessibility of control-related thoughts increases, and a temporary state or sense of (un)controllability is evoked. However, difficulties in replicating some research findings have raised questions about the transferability of these procedures (Cesario, 2014). We also asked more basic questions: What kinds of memories and experiences are activated when people are asked to recall events involving (no) control? Are we really activating only the construct of control, or are we also activating a set of different constructs that researchers do not often consider? Our findings suggest that these different procedures evoke a range of experiences, which could impact the measurement and interpretation of outcome variables.

### What do we manipulate when using recall-based experimental procedures?

Recall-based procedures, often referred to as mindset-priming procedures in social cognitive research, are frequently used in experimental studies without first determining whether they effectively activate the intended psychological construct or multiple different constructs. Chester and Lasko (2021) recently argued that experimental manipulations of psychological constructs may not actually influence their intended mental processes. Therefore, it is important to not only consider "What is retrieved from memory?", but also "How are the memories retrieved?". For example, research on autobiographical memories has shown that the phenomenological characteristics of memories can vary depending on how they were retrieved (Barzykowski & Staugaard, 2016, 2018; Harris et al., 2015). Furthermore, Lammers et al. (2017) found that the difficulty of retrieval can negate or even reverse the effects of recall-based power manipulations. These lines of research suggest that the success of recall-based procedures as experimental tools depends not only on the construct of control itself, but also on the experience of retrieval, including factors such as the difficulty of the recall and the clarity of the memories. Therefore, we tested (a) whether the most commonly used procedures to induce a sense of control effectively tap into the nomological network of personal control, (b) whether these procedures generate comparable experimental conditions when activating memories related to high vs. low control, and (c) how experiences related to control vary between the most commonly used versions of recall-based experimental procedures to induce experiences of controllability and uncontrollability.

The aim of our study was to answer questions about the effectiveness and characteristics of three different types of recall procedures: the free recall procedure (Whitson & Galinsky, 2008), the positive events recall procedure (Kay et al., 2008), and the negative events recall procedure (Rutjens et al., 2010). We analyzed four different datasets, including two re-analyses of existing data and one experimental study and found that the free recall procedure activated strong emotional states, particularly aversive ones in the low control condition. Participants also reported feeling more tired after recalling lack of control events. In addition, a significant percentage of participants in the low control condition displayed active coping attempts, which resulted in a positive ending for the recalled event up to one third of the time, potentially boosting their sense of control. It appears that the free recall control manipulation mainly affects control based on the consequences of one's actions, often referred to as self-efficacy expectations or response-outcome expectations (Bandura, 1977a; Skinner, 1996). These states are prone to produce feelings of helplessness, which are associated with strong aversive emotional reactions (Seligman, 1975).

The negative events recall procedure also revealed troubling results. While it activated negative memories in both low and high control conditions, allowing comparability of effects in terms of valence, the two conditions differed critically in terms of the quality of the memories and other phenomenological aspects of the memories. Asking participants to think about negative controllable events was especially mentally demanding, probably contributing to the problem of selective dropout (Potoczek et al., 2022). This effect could lead to possible confounds as memories that are difficult to retrieve can lower feelings of control and thereby influence sensitive outcome measures (Lammers et al., 2017).

By inducing mainly positive emotions related to the state of having control (or not), the positive events recall manipulation (Kay et al., 2008) efficiently resolved the problem of inducing different emotional states in high and low control conditions. However, it also differentially manipulated the intra- vs. interpersonal aspect of the memory, the specificity of the memory (more specific events for low control), and temporality of the memory (briefer events for low control). Critically, the second experimental study revealed that general perceptions of personal control over one’s life and control over life’s outcomes (self-mastery) were not affected by the positive event recall manipulation. It seems that this procedure activates a different aspect of control than the free recall and the negative events recall procedures. It is not related to perceptions of efficacy, agency, or competency, but rather to the perception of predictability vs. randomness of different events that happen in life, or uncertainty about the world (Whitson et al., 2015).

To be clear, that the positive recall manipulation emphasizes specific aspects of personal control is not necessarily a problem. Still, when using this manipulation to replicate effects obtained with the free recall or negative events recall procedure, it should not be surprising that different types of manipulations might yield different results even on similar outcome measures. For example, the positive recall manipulation by activating a view of the reality and one’s actions as driven by randomness, it might lead for a search for order and predictability (e.g., by believing in a controlling god, a benevolent governmental system, or support for hierarchical structures in organizations; Friesen et al., 2014; Kay et al., 2008). In contrast, the free recall or negative events recall control manipulation by activating the image of oneself as inefficient or incompetent, it might induce the need to boost one’s positive self-view and to reaffirm the agency of oneself or the ingroup, (e.g., by enhancing ingroup-bias, blaming others, or search for conspiracy theories; Bukowski, de Lemus, et al., 2017﻿; Fritsche et al., 2013; Kofta et al., 2020; Whitson & Galinsky, 2008). Thus, researchers might face problems if they use a different procedure to the one used in the research they are attempting to replicate. A failed replication of this type (which in fact, cannot be treated as a direct replication) might lead to a conclusion that the effect is weak or non-existent whereas it might be restricted only to certain types of experimental conditions.

To sum up, our findings show that three different types of recall-based procedures, which mainly focus on differences in the valence of the recalled events, also differently shape the experience of control, effortfulness related to the recall process, and phenomenological characteristics of the event memory. In fact, the manipulations differ not only in valence but also in the kind of task the participants receive, so for example, in the negative events recall procedure participants are instructed to think more but also more prospectively. Importantly, different experimental procedures might activate different constructs of control, which can activate different responses to uncontrollability.

### Mapping the construct of personal control

How do the recall-based control manipulations map into the construct of personal control? One common aspect of control manipulations is that they all, to a different degree, activate autobiographical memories, meaning episodes and experiences that directly involve the self and reflect the self as agentic and able to influence the environment in a desired direction. However, under the surface of using the same (recall-based) type of procedure to evoke perceptions and feelings of control or its lack, different procedures, by differently impacting how people experience a particular recalled situation or event, can also activate diverse constructs of control. For example, some recall-based experimental manipulations by activating personal uncertainty, related to one’s perceived abilities to control an unpredictable environment, can also boost the need for structure, which is a common finding in the compensatory control literature (Ma & Kay, 2017; Whitson & Galinsky, 2008). Consistent with this interpretation, emotions associated with uncertainty about the world specifically drive the effects of compensatory control (Whitson et al., 2015). Other types of control manipulations can affect a different aspect of personal control, which is based on the effectiveness of specific actions in achieving desired outcomes (i.e., means – ends relations; Bandura, 1977a; Skinner, 1996). Thus, compensatory control effects might not appear when a self-efficacy aspect of control is manipulated using procedures that directly affect the perception of action – outcome non-contingency (e.g., van Elk & Lodder, 2018). Thus, interchanging procedures that are thought to invariably manipulate the constructs of uncertainty and lack of control might actually lead to a host of variable effects and contribute to difficulties in replicating some reported effects. It seems that the field of research on personal control is still lacking sufficient direct replication attempts to rule out the possibility that construct validity issues importantly contribute to the lack of coherence in the obtained results (still some experimental procedures are used interchangeably thus allowing only for conceptual replications). However, construct validation should not be considered as the only or main reason of replication failures in psychological research as there are several other factors that contribute to this problem, such as publication biases, questionable research practices etc. (see Pashler & Wagenmakers, 2012).

Additionally, the specific types of experiences related to the recall of autobiographical memories (e.g., its affective valence) can shape the type of reactions to uncontrollability. For example, recall of negative, frustrating episodes that affect one’s self-esteem can enhance defensive and avoidance-based types of responses, whereas recall of positive events might activate responses that restore a view of the world as an ordered and predictable place. A remaining question is whether these ways of inducing control threat are comparable to states of uncontrollability that people experience on an everyday basis (e.g., due to long lasting unemployment, illness etc.). In other words, is the control construct that is manipulated via recall-based procedures equivalent to other experiences of (lack of) control?

Our results revealed that participants scored lower on event-related sense of control measures after inducing lack of control for all three recall-based manipulations. However, we found lower scores for more generalized perceptions of control (e.g., self-mastery), only in the free- and negative-recall control manipulations. Also, the only positive-events recall procedure differs substantially from both the free-recall and negative event recall manipulations, revealing lower scores on outcome measures that assess fatigue related to the recall and effortfulness of coping with the recalled situation. In sum, there is a need to test the effects of different control manipulations on the same outcome variables in more depth, especially those variables that were widely used in previous research (such as need for structure, belief in conspiracies etc.). This way we could account for the specific reasons (mechanisms) that a particular replication did not work. The research presented here was meant to set up a methodological ground for such future replication types of research.

### Recommendations for future research on personal control

Different experimental procedures evoke the construct of control to different degrees; they sometimes even relate more to other constructs than to control. Thus, we need to reconsider what we are really manipulating when we think that we are inducing a given psychological state like a lack of personal control. What other sources of variability are there when using different procedures to activate the same construct? Based on our research, it seems that the use of different recall-based procedures can also lead to the activation of different mental constructs or representations of personal control and other phenomenological states associated with it. Importantly, this issue is not specific to this field of research on control motivation. For example, research on self-control and executive function suffer from some of the same issues (Inzlicht & Berkman, 2015; Lurquin & Miyake, 2017), suggesting that those fields also need more precise approaches to define and operationalize their variables of interest.

How can we overcome these problems and move the field forward? To improve the study of control constructs, several recommendations can be made. First, clear operational definitions of the construct of control should be established, specifying the different types of control such as self-efficacy, generalized control beliefs, competence, autonomy, etc. (Bandura, 1997b; Skinner, 1996). Second, independent validations of control manipulations are needed, using a variety of methods such as recall-based, imaginary, experience-based, and phenomenological-based methods. Lurquin and Miyake (2017) made this suggestion for research on self-control, but it is equally valid for research on personal control. Additionally, using validated measures of personal control as a dependent variable and comparing the results with different types of experimental manipulations can provide a stronger test for the construct validity of a given experimental procedure. Third, it is important to probe the effects of the manipulations applied, by assessing different outcome measures and carefully choosing manipulation checks (see Fiedler et al., 2021). Content-based analyses can also provide a valid test for manipulations that involve participants reflecting on their own experiences and memories. This approach might be especially promising, as some researchers even argue that manipulation checks (e.g., asking participants about their momentary feelings of control) can decrease the effectiveness of experimental manipulations (Hauser et al., 2018).

Finally, it is also important to consider the context- and domain-specificity of control manipulations. For instance, recent findings indicate that domain-specific control threat (related to the COVID-19 pandemic) predicts endorsement of specific conspiracy beliefs (Stojanov et al., 2023). This aligns with some theoretical perspectives highlighting the crucial role of a match between the control-threat domain and a domain in which specific (collective) actions to restore control can be taken (e.g., the domain of ecological threat; Potoczek et al., 2022). Moreover, contextual factors such as the culture in which participants are embedded in, can activate different models of self and agency, thereby influencing the very experience of control and how individuals respond to recall-based control threat manipulations (Gibbs et al., 2023). Thus, it seems important to focus more closely on the contextual and domain specific aspects of experimental manipulations of control, particularly when attempting to replicate existing research findings.

These methodological reflections go beyond research on control. We propose that we ought to pay closer attention to the definitions of the construct studied, that it is important to carefully use recall-based manipulations as a way to activate different psychological constructs (e.g., goals, emotions, mental constructs, autobiographical memories, etc.), and that there is a need to assess to what extent these concerns regarding construct validation of experimental methods might restrict our abilities to generalize certain empirical findings.

Instead of providing ready-made solutions, which often turn out to be oversimplified, our aim here was to draw the attention of researchers in different fields of experimental psychology to the need to know what we are manipulating and measuring. It is only by doing so that we can gain a better understanding of our results, give reliable advice, and build a cumulative science.

## Footnotes

1 The datasets for all studies described in this manuscript and pre-registration forms are available in the OSF repository: https://osf.io/mk7s2/?view_only=c09801257465477493d7364887d030ee.

2 It must be acknowledged that these two contexts, although relevant for participants of this study, are not equally broad, the social context encompassing more types of situations.

3 We have made efforts to maintain the original meaning of this manipulation when translating it but also to adjust it to the language of the study, so the instruction would read natural. The German language version was adapted (and pre-tested) in the following form: “Please try to think of something positive that happened to you in the past few months and that you brought about yourself (i.e., that you were able to influence)”.

4 The measures applied in this experiment were pre-tested in an additional study, in which only the free recall procedure was applied. We report all the results obtained in this study in the Supplementary Materials, available here: https://osf.io/mk7s2/?view_only=c09801257465477493d7364887d030ee.

5 The number of participants in each condition was as follows: Free recall / low control: 67, Free recall / high control: 60, Positive events recall / low control: 70, Positive events recall / high control: 89, Negative events recall / low control: 67, Negative events recall / high control: 42, Baseline: 111.

6 As in Study 1b, we have adjusted the translation to the language of the study. The translation into Polish reads (after back translation): “Please try and think of something positive that happened to you in the past few months that was [not] caused by yourself (i.e., that you had [absolutely no] control over.)”

7 Examples of controllable and uncontrollable experiences for each of the types of manipulations: Uncontrollability / Free recall: Car accident, disease of a family member. Uncontrollability / Positive-events recall: Going on a surprise trip with a spouse, unexpected raise of salary. Uncontrollability / Negative-events recall: Being left by a partner, argument at work. Controllability / Free recall: Helping one’s child in a homework, solving important problem at work. Controllability / Positive-events recall: Accepting a proposal from fiancé, planning holidays. Controllability / Negative-events recall: Refusing a loan to a friend, taking painkillers when being sick. Baseline: Describing the route from home to work, from school to shop etc.

8 This study was pre-registered and the document is available here: https://aspredicted.org/M61_CKZ

9 We have not included the baseline into the ANOVA design as planned in the pre-registration since it would be not possible to clearly interpret the results of such an analysis. Thus, we used a 2 x 3 design and additionally compared the results for high and low control conditions with the baseline condition.

10 An additional CFA analysis performed to check for the distinctiveness of the applied measures of effortfulness showed that a model distinguishing between the two applied measures has a relatively poor fit, which means that they could build one factor. Still, we report the findings for both measures separately as on a theoretical level they assess different aspects of fatigue, and the specific results for the fatigue during recall and difficulty of retrieval measures might be informative for the reader.

11 There was a significant three-way interaction with gender of participants for the clarity of the memory (*F*(2,383) = 4.126, *p* = .017, *η*^*2*^ = .02) revealing different response patterns for women and men in the free recall and negative recall procedures. This was the only significant interaction with gender for all out of the eleven outcome variables.
